# Spatial-temporal evolution and peak prediction of embodied carbon emissions in China's interregional trade

**DOI:** 10.3389/fpubh.2022.1010521

**Published:** 2022-10-20

**Authors:** Shu Mo, Ting Wang

**Affiliations:** ^1^School of Management, Guizhou University, Guiyang, China; ^2^Guizhou Provincial Key Laboratory of Internet Plus Intelligent Manufacturing, Guiyang, China; ^3^Baltic Sea Region Research Center, Guizhou University, Guiyang, China

**Keywords:** embodied carbon emissions, spatial-temporal evolution, multi-regional input-output model, Kuznets curve, peak prediction, economic growth, environmental pollution

## Abstract

The embodied carbon in inter-regional trade has a vital impact on the allocation of carbon emission reduction obligations and the formulation of carbon emission reduction strategies. Fewer studies have examined the spatial-temporal evolution pattern and peak prediction of embodied carbon emissions in China's inter-regional trade compared with the more numerous results on embodied carbon in international commerce. This paper applies the multi-regional input-output method to estimate the embodied carbon in inter-regional trade resulting from value-added transfer and investigates the spatial and temporal evolution of its patterns. The existence of an environmental Kuznets curve model with embodied carbon emissions as the index of environmental pollution in China is examined, and the time of the inflection point is calculated. The environmental Kuznets curve model is divided into four stages, and a two-dimensional model of economic development and embodied carbon emissions is proposed. The empirical findings indicate that the embodied carbon in China's interregional commerce has an overall rising tendency in the temporal dimension and a distribution characteristic of high in the west and north and low in the east and south in the spatial dimension. The Environmental Kuznets curve, which uses embodied carbon emissions as a measure of environmental pollution, has an inverse U-shaped and the time required to reach the inflection point varies by area. Economic development cannot be cross-stage but can shorten the duration of high carbon emissions. The government should promote the development of differentiated carbon emission reduction policies in each region, construct an inter-regional cooperative carbon emission reduction mechanism, encourage the low-carbon development of inter-regional trade, and realize the internal cycle of China's green economy. This study serves as a guide for the regions to establish scientific and acceptable carbon emission reduction strategies in order to achieve quality interregional trade development.

## Introduction

According to the International Energy Agency, China is the top carbon dioxide emitter in the world (1). In this regard, the Chinese government has consistently mentioned China's 2030 carbon peak objective, its 2060 carbon-neutral vision, and new steps to boost the country's independent contribution during the 75th UN General Assembly general discussion, the Climate Ambition Summit co-organized by the UN and relevant national activities (2). On the one hand, this demonstrates that China places a high priority on ecological and environmental issues. On the other hand, worldwide public opinion pressure and green trade obstacles in China's external environment provide a severe development obstacle. In light of the issue's complexity, it is imperative to establish a low-carbon economy conducive to attaining economic and environmental efficiency.

To establish a low-carbon economy, each region must take unique steps to reduce its local CO2 emissions. Nevertheless, due to China's unequal regional growth, regional carbon reduction objectives will differ. Before formulating regional emission reduction policies, two considerations must be made: China's inter-regional trade is frequent, and the carbon transfer embodied in inter-regional trade has a significant impact on regional carbon emissions (3); Moreover, will the embodied carbon emissions in inter-regional trade continue to increase with economic growth? Or will there be a turning point for it? Therefore, embodied carbon emissions in inter-regional trade impact not only the definition of carbon reduction duty in various regions of China but also the attainment of carbon peaking by 2030 in China. As a result, the focus of this paper will be on embodied carbon emissions in inter-regional trade as the primary research object.

There are currently three primary types of relevant literature related to this paper. The first category of literature examines the connection between economic growth and environmental pollution. Some academics contend that economic progress presupposes environmental contamination (4). Nonetheless, many academics agree that economic growth is a practical approach to supporting environmental conservation (5–7). In the 1950s, Kuznets introduced the inverted U curve theory for per capita income and GDP (8). Grossman and Krueger inherited and developed Kuznets' famous inverted U curve hypothesis by proposing the environmental Kuznets curve, which indicates that the relationship between economic development and environmental pollution is an inverted U (9). Environmental pollution is low at the beginning of economic development, increases with economic development, but decreases when economic development reaches its a certain stage. Numerous research has supported this viewpoint (10–13). Despite this, there remain debates over the environmental Kuznets curve. Some academics have presented other form theories (e.g., N-shaped, inverted N-shaped, etc. (14–16), and a few academics have even questioned the existence of the environmental Kuznets curve (17).

The second kind of literature investigates embodied carbon emissions from an inter-regional trade perspective. China is an enormous territory with a vast market space, and each region develops its economy autonomously, with varying resources and technology (18). Regional variations exist in the division of labor within the value chain. The growing separation of production and consumption locations contributes to the flourishing growth of regional commerce (19). Concurrently, this results in the progressive growth of trade-related carbon emissions. In the national division of labor system, economically developed regions control low-energy-consuming, low-emission, and high-value-added production processes, while outsourcing high-energy-consuming, high-emission, and low-value-added production processes to economically developing regions, thereby gaining enormous economic benefits, and to some extent, avoiding some producer responsibility for pollution emissions (20, 21). Behind the limited value-added trade gains of developing regions is immense pressure to reduce carbon emissions, even becoming a pollution refuge for economically developed regions, thereby aggravating the inequity of carbon emission reduction rights and responsibilities in China (22–24).

The third category of literature is about the research on the measurement methods of embodied carbon emission in trade. There are two primary methodologies for assessing embodied carbon in inter-regional trade. The life cycle assessment approach evaluates the environmental effects of a product from the input of raw materials to the output of a product (25), such as the carbon footprint of the agricultural industry, the building industry, and other industries (26–28). The input-output method employs the entire economic system as the research object and investigates the input-output linkages between various industrial sectors. And a single-region input-output table employs to calculate the carbon footprint of the supply side and the consumption side of the entire production process (29, 30), or the local carbon emission intensity (31, 32). The multi-regional input-output method, on the other hand, explores the economic ties between regions by examining the interconnections between regions (33–36). Since the emissions and transfers of trade embodied carbon is global and mobile, numerous researchers have combined the multi-regional input-output approach with econometric methods to establish the trade embodied carbon at different scales among Chinese provinces and industries, or countries around the world, in order to analyze their overall and individual characteristics (37–45).

Although previous research on embodied carbon emissions in inter-regional trade is valuable for the rational distribution of regional emission reduction duties. Yet, it is also not difficult to discover that there is very little pertinent research on embodied carbon emissions in inter-regional trade inside China in comparison to the numerous studies on embodied carbon emissions from international trade. Furthermore, there is hardly any research that examines the relationship between embodied carbon emissions in inter-regional trade and economic growth and the peak potential of embodied carbon emissions in inter-regional trade within the agreed-upon time range. Based on this, the following are the potential marginal contributions of this research. First, this paper attempts to measure embodied carbon emissions in inter-regional trade and analyze its spatial and temporal evolution pattern from the perspective of value added by using the newly released regional in-put-output tables in China, which can reflect more realistically than previous studies on the economic links and environmental impacts of different regions in China in the trade process. Secondly, this paper will analyze the relationship between embodied carbon emissions and economic development in China, test whether the Kuznets curve hypothesis of embodied carbon emissions in interregional trade exists for China as a whole and each province, and examine the time required for various regions to reach the inflection point of embodied carbon emissions in interregional trade. Thirdly, this paper stages the environmental Kuznets curve model and presents a two-dimensional model of economic development and embodied carbon emissions in interregional trade, which deepens the relevant environmental economics research.

This research is organized as follows. Section Methods and data describes the primary methodology and data source. Section Spatial and temporal patterns of embodied emissions illustrates the geographical and chronological trends as well as the dynamic evolution of embodied carbon emissions in China's interregional trade. The empirical results of the analysis are illustrated in Section Empirical analysis, and the link between economic growth and embodied carbon emissions in interregional trade is further explored in Section Discussion of the Kuznets curve based on embodied carbon. Section Conclusions and policy recommendations concludes finally.

## Methods and data

### Trade embodied carbon measure

A multi-regional input-output model is used to assess the embodied carbon of China's intra-regional trade, and the table of China's regional input-output for S provinces is given in Table 1.

**Table 1:** Multi-regional input-output tables for China.

		**Intermediate use**				**Final demand**				**Export**	**Total output**
		**1**	**2**	**…**	**S**	**1**	**2**	**…**	**S**		
Intermediate inputs	1	U_11_	U_12_	…	U_1S_	D_11_	D_12_	…	D_1S_	EX_1_	X_1_
	2	U_21_	U_22_	…	U_2S_	D_21_	D_22_	…	D_2S_	EX_2_	X_2_
	…	…	…	…	…	…	…	…	…	…	…
	S	U_S1_	U_S2_	…	U_SS_	D_S1_	D_S2_	…	D_SS_	EX_SS_	X_S_
	Import	IM_1_	IM_2_	…	IM_S_	DIM_1_	DIM_2_	…	DIM_S_		
Value added		Va_1_	Va_2_	…	Va_S_						
Total input		(X_1_)′	(X_2_)′	…	(X_S_)′						

U_ij_ and D_ij_ denote the demand matrices for intermediate use and final consumption in region j for region i exported to region j, respectively. IM_i_, Va_i_ and (X_i_)' denote the matrix of imports of intermediate goods, the vector of value-added, and the vector of total inputs for region i, respectively. EX_i_, DIM_i_, and X_i_ denote the export vector, the final product import vector, and the total output vector for region i respectively. The specific input-output model is as follows:


(1) $$\begin{array}{l} \left[\begin{array}{c} X_{1}\\ X_{2}\\ \ldots \\ X_{S} \end{array}\right]=\left[\begin{array}{cccc} A_{11} & A_{12} & \ldots & A_{1S}\\ A_{21} & A_{22} & \ldots & A_{2S}\\ \ldots & \ldots & \ldots & \ldots \\ A_{S1} & A_{S2} & \ldots & A_{SS} \end{array}\right]\left[\begin{array}{c} X_{1}\\ X_{2}\\ \ldots \\ X_{S} \end{array}\right]+\left[\begin{array}{c} D_{1}\\ D_{2}\\ \ldots \\ D_{S} \end{array}\right] \end{array}$$

A_ij_ (i ≠ j) is the matrix of coefficients of demand for intermediate goods from region j to region i, and A_ii_ is the matrix of direct coefficients for region i. Rectification gives:


(2) $$\begin{array}{l} \left[\begin{array}{cccc} X_{11} & X_{12} & \ldots & X_{1S}\\ X_{21} & X_{21} & \ldots & X_{2S}\\ \ldots & \ldots & \ldots & \ldots \\ X_{S1} & X_{S2} & \ldots & X_{SS} \end{array}\right]=\left[\begin{array}{cccc} B_{11} & B_{12} & \ldots & B_{1S}\\ B_{21} & B_{21} & \ldots & B_{2S}\\ \ldots & \ldots & \ldots & \ldots \\ B_{S1} & B_{S2} & \ldots & B_{SS} \end{array}\right]\left[\begin{array}{cccc} D_{11} & D_{12} & \ldots & D_{1S}\\ D_{21} & D_{21} & \ldots & D_{2S}\\ \ldots & \ldots & \ldots & \ldots \\ D_{S1} & D_{S2} & \ldots & D_{SS} \end{array}\right] \end{array}$$

(3) $$\begin{array}{l} \left[\begin{array}{cccc} B_{11} & B_{12} & \ldots & B_{1S}\\ B_{21} & B_{21} & \ldots & B_{2S}\\ \ldots & \ldots & \ldots & \ldots \\ B_{S1} & B_{S2} & \ldots & B_{SS} \end{array}\right]={\left[\begin{array}{cccc} I-A_{11} & -A_{12} & \ldots & -A_{1S}\\ -A_{21} & I-A_{21} & \ldots & -A_{2S}\\ \ldots & \ldots & \ldots & \ldots \\ {-A}_{S1} & {-A}_{S2} & \ldots & I-A_{SS} \end{array}\right]}^{-1} \end{array}$$

Where B is the Leontief inverse matrix. From Equation 3, it follows that:


(4) $$X_{j}=\sum_{m=1}^{S}(B_{jm}\sum_{t\neq i}^{S}B_{mt})$$

Further obtained:


(5) $$X_{ij}=L_{ii}A_{ij}X_{j}=L_{ii}A_{ij}\sum_{m=1}^{S}(B_{jm}\sum_{t\neq i}^{S}B_{mt})$$

L_ii_ is the Leontief inverse matrix of region i.


(6) $$\begin{array}{l} X_{i}=\overset{S}{\underset{j=1}{\sum }}X_{ij}=X_{ii}+\overset{S}{\underset{j=1,j\neq i}{\sum }}X_{ij} \end{array}$$

Substituting Equation 5 into Equation 6 and equation 4 yields:


(7) $$\begin{array}{l} \begin{array}{l} X_{i}=L_{ii}D_{ii}+L_{ii}\overset{S}{\underset{i=1,j\neq i}{\sum }}D_{ij}+L_{ii}\overset{S}{\underset{j=1,j\neq i}{\sum }}A_{ij}X_{j}\\ \;=L_{ii}D_{ii}+L_{ii}\overset{S}{\underset{i=1,j\neq i}{\sum }}D_{ij}+L_{ii}\overset{S}{\underset{j=1,j\neq i}{\sum }}A_{ij}L_{jj}D_{jj} \end{array}\;\\ \;\begin{array}{ll} + & L_{ii}\overset{S}{\underset{t=1,j\neq i}{\sum^{\text{​}}}}A_{ij}\overset{S}{\underset{m=1}{\sum^{\text{​}}}}B_{jm}D_{mi} \end{array}\\ \;\begin{array}{ll} + & L_{ii}\overset{S}{\underset{j=1,j\neq i}{\sum^{\text{​}}}}A_{ij}\overset{S}{\underset{m=1}{\sum^{\text{​}}}}B_{tm}D_{mj}L_{ii}\overset{S}{\underset{j=1,j\neq i}{\sum^{\text{​}}}}A_{ij}L_{jj}D_{jj}\\ + & L_{ii}\overset{S}{\underset{t=1,j\neq i}{\sum^{\text{​}}}}A_{ij}\overset{S}{\underset{m=1}{\sum^{\text{​}}}}B_{jm}\overset{S}{\underset{t\neq i}{\sum^{\text{​}}}}D_{mt}+\delta \end{array} \end{array}$$

Defining the diagonal matrix of carbon emission factors for CO_2_ as F_s_, both sides of Equation 7 are multiplied by F_s_ simultaneously to obtain the decomposition formula for CO_2_ emissions from province i as follows.


(8) $$\begin{array}{l} (C_{i})'=F_{i}L_{ii}D_{ii}+F_{i}L_{ii}\sum_{i=1,j\neq i}^{S}D_{ij}+F_{i}L_{ii}\sum_{j=1,j\neq i}^{S}A_{ij}L_{jj}D_{jj}\\ \;+\;F_{i}L_{ii}\sum_{t=1,j\neq i}^{S}A_{ij}\sum_{m=1}^{S}B_{jm}D_{mi}\;\\ \;+\;F_{i}L_{ii}\sum_{j=1,j\neq i}^{S}A_{ij}(\sum_{m=1}^{S}B_{tm}D_{mj}-A_{ij}L_{jj}D_{jj})\\ \;+\;F_{i}L_{ii}\sum_{j=1,j\neq i}^{S}A_{ij}\sum_{m=1}^{S}B_{jm}D_{mi}+\delta \; \end{array}$$

The first term indicates that embodied carbon dioxide is included in the final product used in province i. The second term indicates that carbon dioxide is embodied in the final product produced in province i being exported to province j to meet the final demand in province j. The third term indicates that carbon dioxide is embodied in exporting intermediate products from province i to province j for input into production as intermediate products in province j. The fourth term indicates that the intermediate product embodied by CO_2_ in province i is exported and re-imported to be consumed in province i. The fifth term indicates that the intermediate product of carbon dioxide embodied in province i is imported into province j after two transfers. The sixth term indicates that the intermediate product of carbon dioxide embodied in province i is exported twice and then finally used in a third province t. The sixth term indicates that the intermediate product of carbon dioxide embodied in province i is exported twice and then finally used in province t. The seventh term denotes the export sector outside province i. This sector is not considered as this paper only examines carbon embodied in China's inter-provincial trade. Those involving inter-regional carbon transfers include the second, third, and fifth terms. The embodied carbon emissions from intermediate goods and final consumption supplied by province i in the end:


(9) $$\begin{array}{l} EC_{ij}=F_{i}L_{ii}A_{ij}\sum_{m=1}^{S}B_{tm}D_{mj}+F_{i}L_{ii}D_{ii}\\ \;+F_{i}L_{ii}A_{ij}(L_{jj}D_{jj}L_{jj}D_{jj}-A_{ij}L_{jj}D_{jj}) \end{array}$$

EC_ij_ is the carbon dioxide emissions generated by province i to produce intermediate and final products exported to province j.

### Environmental Kuznets curve model

Grossman and Krueger put forward the environmental Kuznets curve hypothesis, which suggests that the degree of environmental pollution and economic growth show a tendency to grow first and then decrease, namely, the relationship between the two shows an inverted U-shape. To illustrate graphically, if GDP per capita is the horizontal coordinate and the environmental pollution level is the vertical coordinate, the environmental pollution level shows a shape of an inverted U-shaped curve that increases first and then decreases with income growth.

The relationship between carbon emissions and economic growth has been studied from the perspective of the environmental Kuznets curve in a large amount of literature (46, 47). Nevertheless, there is a lack of studies on the relationship between embodied carbon emissions and economic growth due to regional trade in the context of the increasing developmental linkages and the close inter-regional trade of inputs and outputs in China. Embodied carbon emissions in inter-regional trade refers to the direct and indirect carbon dioxide emissions implicitly generated throughout the production chain of products and services, which is an important but more easily ignored environmental pollution variable (48).

Hence, a logistic regression model of economic development and environmental pollution is constructed utilizing the environmental Kuznets curve concept. In this paper, environmental pollution is represented by embodied carbon emissions in inter-regional trade, which can provide a more comprehensive measure of the extent of environmental pollution in a region than carbon emissions. Economic growth is represented by GDP per capita due to GDP per capita excluding the population factor is a better measure of the economic growth level of a region than the aggregate indicator.


(10) $$\begin{array}{l} LnEC_{st}=\alpha {\left(LnPGDP_{st}\right)}^{2}+\beta LnPGDP_{st}+\eta +\lambda_{s}+\mu_{st} \end{array}$$

Subscript s denotes Chinese provinces, and t denotes the year. EC represents embodied carbon in inter-regional trade; PGDP represents real GDP per capita in constant 1,978 prices; η is the intercept that does not vary with individuals; λ_s_ is the individual effect; α and β are parameters to be estimated; μ_st_ is the random error term.

When α = 0 and β≠0, the relationship between embodied carbon in inter-regional trade and economic development is linear; when α < 0 and β > 0, the relationship between embodied carbon in inter-regional trade and economic development is inverted U-shaped; when α > 0 and β < 0, the relationship between embodied carbon in inter-regional trade emissions and economic development is U-shaped.

### Data description

The database for the multi-regional input-output model was taken from the China Carbon Accounting Database for multi-regional input-output tables of China in 2002, 2007, 2012, 2015, and 2017. Since the production technology structure of a province re-mains stable over the short term, the input-output table data for each province in 2002 were utilized throughout the period 2001–2006. For 2007–2011, each province's 2007 in-put-output table data is utilized for calculating purposes. For 2012–2014, the statistics from each province's 2012 input-output tables are used. 2015–2016 is derived using each province's 2015 input-output table data. 2017–2019 is calculated using each province's in-put-output table data from 2017.

The fossil energy CO_2_ emission factors were determined using the methods described in the United Nations Intergovernmental Panel on Climate Change's 2006 Guidelines for National Greenhouse Gas Emission Inventories. The information on fossil energy consumption and combustion emissions was gathered from the statistics yearbooks of each province and city (autonomous area) for the relevant year and the China Energy Statistical Yearbook.

Tibet, Hong Kong, Macau, and Taiwan are not considered in this research due to a lack of data, and panel data of 30 Chinese provinces from 2001 to 2019 are utilized as the empirical study's sample. The national energy consumption, gross domestic product, and gross regional product are taken from the China Statistical Yearbook. The population size is collected from the China Statistical Yearbook of Population and Employment.

Logarithms are used for the aggregate indicators in this article. Adopting a logarithmic form can significantly minimize the disparities between the samples and bring their distribution closer to a normal distribution, which is favorable to fulfilling the regression's hypothesis requirements. In addition, the dependent variable has a logarithmic shape. Hence logarithmic data are chosen for the independent variables as well.

## Spatial and temporal patterns of embodied emissions

From 2001 to 2019, there was an increasing trend in the total embodied carbon emissions in inter-regional trade. As the standard of living has grown as a result of China's fast economic development, expenditure and consumption have risen. At the same time, the adoption of an investment-driven growth strategy and the active participation of the Chinese government have increased in significance. These two factors together have considerably improved investment and government consumption. Embodied carbon emissions in inter-regional trade have increased dramatically based on this economic development scenario and consumption pattern. Huge embodied carbon emissions of the transfer reflect the tight economic linkages between China's regions.

Furthermore, compared to the prior time, the growth rate from 2015 to 2019 is much lower and shows noticeably wider regional disparities. Embodied carbon emissions in inter-regional trade primarily flow from the eastern region which is economically developed to the center and western regions which are economically underdeveloped. As a result of the Western Development Strategy's execution, the northwest Chinese provinces of Inner Mongolia and Shaanxi have progressively grown into significant hubs for the export of energy. Massive economic growth and fast urbanization have resulted from the large-scale expansion of energy in these areas, but it has also brought about a number of issues, including ecological and environmental harm.

In terms of the spatial dimension, the spatial pattern of China's embodied carbon emissions in inter-regional trade is high in the west and low in the east, as well as high in the north and low in the south, with the Northwest, central, and northeast having the highest embodied carbon emissions. As can be seen, regional disparities between economic growth and environmental pollution are expanding, whereas regional differences in economic aggregates are diminishing. In 2001, Central, Northwestern, and northeastern areas had the most significant embodied carbon emissions. In 2007, the center area, the Northwest, and the southwest had the most significant embodied carbon emissions, in that order. In 2013, the northwestern, central, and northern coastal areas had the most significant embodied carbon emissions resulting from the value-added transfer. By 2019, the western had the most significant embodied carbon emissions, while the central region had a decline in embodied carbon emissions.

**Figure 1:**
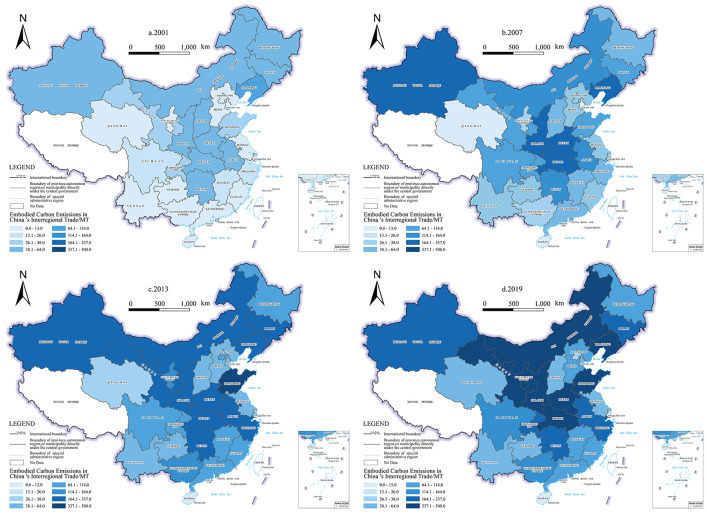
Spatial and temporal patterns of embodied carbon emissions in inter-regional trade.

From 2001 to 2019, the pattern of embodied carbon emissions in inter-regional trade transfer across regions in China had altered significantly (as shown in Figure 1). In 2001, the Northeast area has a tighter carbon link with the Northern and Eastern coastal regions and the Central region. Hence, embodied carbon emissions in the central region more than in the northern coastal and eastern regions. In 2007, 2013, and 2019, the central region's embodied carbon emissions exhibit a two-way pattern, absorbing a considerable number of export-based emissions from the eastern coast and transmitting a large number of consumption-based emissions to the northwest and other regions. In addition, embodied carbon emissions in inter-regional trade transfers from the Northwest to other regions are rising. Northeast area embodied carbon emissions exhibit a declining trend. In contrast, embodied carbon emissions in inter-regional trade from the eastern coastal and northwest regions rise. The exchange suggested that carbon emissions from the coastal and central areas of the south are on the decline.

Regional differences in embodied carbon emissions are strongly correlated with the decline in prices of energy resources and energy-intensive industrial products such as steel and petrochemicals in China following the 2008 financial crisis, as well as the window period for regional development strategies such as the Western Development. The financial crisis in 2008 had a negative impact on the industrial sector, which in turn damaged fundamental industries such as coal and steel. Due to the increased rigidity of pro-duction in the coal, steel, and iron sectors, stocks tend to rise, resulting in a bigger effect on their pricing. And this price adjustment is extremely detrimental to the economic growth of places that mostly export energy and raw materials, while the impact on regions that primarily produce industry is somewhat less severe. This transfer of value among various businesses is the cause of geographical variations in embodied carbon emissions.

## Empirical analysis

### Panel unit root test

It is necessary to conduct a unit root test on the panel data to determine the smooth-ness of the data prior to doing cointegration on the panel data because cointegration be-tween variables presupposes that the variables be homogenous and single-integer. The most prevalent tests for determining the unit root of panel data are the LLC test, Breitun test, IPS test, Fisher test (including ADF Fisher and PP Fisher test), and Hadri test. In this study, the unit root test was conducted using six techniques, and the findings are presented in Table 2.

**Table 2:** Panel unit root test results.

**Variable**	**Test**	**Original value**		**First difference**	
		**Constant**	**Constant and trend term**	**Constant**	**Constant and trend term**
LnEC	Breitun		−3.7563 (0.0042)		−8.3214 (0.0000)
	LLC	−0.7962 (0.3415)	−2.1359 (0.0457)	−14.8231 (0.0000)	−13.9403 (0.0000)
	Hadri	8.3196 (0.0000)	6.9721 (0.0000)	2.1309 (0.2771)	9.2138 (0.0000)
	IPS	2.8754 (0.9880)	0.9754 (0.8214)	−9.9718 (0.0000)	−7.5235 (0.0000)
	ADF Fisher	2.2691 (0.9863)	0.9513 (0.8725)	−9.3677 (0.0000)	−6.6956 (0.0000)
	PP Fisher	2.4538 (0.9902)	1.7865 (0.9183)	−9.8914 (0.0000)	−8.1237 (0.0000)
LnPGDP	Breitun		5.2139 (1.0000)		−0.6825 (0.3721)
	LLC	−6.3245 (0.0000)	−2.9821 (0.0086)	−9.7826 (0.0000)	−12.4289 (0.0000)
	Hadri	14.1972 (0.0000)	9.6953 (0.0000)	5.0674 (0.0000)	12.5175 (0.0000)
	IPS	2.4139 (0.8675)	2.5472 (0.8959)	−5.1314 (0.0000)	−4.7863 (0.0009)
	ADF Fisher	1.8967 (0.9012)	2.2833 (0.9197)	−6.4267 (0.0000)	−3.9755 (0.0006)
	PP Fisher	−0.5648 (0.4853)	3.5376 (0.9981)	−5.2783 (0.0000)	−5.6430 (0.0000)
(LnPGDP)^2^	Breitun		5.2413 (1.0000)		−0.3692 (0.4465)
	LLC	−3.4155 (0.0026)	−4.7806 (0.0000)	−9.4276 (0.0000)	−11.4259 (0.0000)
	Hadri	15.7390 (0.0000)	10.6381 (0.0000)	6.5732 (0.0000)	13.5215 (0.0000)
	IPS	4.5821 (0.9751)	0.4625 (0.7835)	−6.3251 (0.0000)	−4.5687 (0.0016)
	ADF Fisher	4.6288 (0.9836)	0.3536 (0.7001)	−6.7189 (0.0000)	4.2752 (0.0009)
	PP Fisher	3.3516 (0.9992)	−0.3942 (0.5470)	−6.3616 (0.0000)	−5.1739 (0.0000)

The concomitant probabilities of the estimated quantities are in parentheses.

The tests on the original values of the variables, whether they contain only the constant term or both the constant and trend terms, indicate, with very few exceptions, that the null hypothesis of the existence of a unit root cannot be rejected. However, when the first-order difference of the variables is tested, the null hypothesis of the existence of a unit root is firmly rejected. Therefore, the panel data for the logarithm of embodied carbon emissions in inter-regional trade, GDP per capita, and GDP per capita squared are first-order single integer.

### Panel cointegration test

According to the findings of the unit root test, every time series in the panel data is found to be a unit root process. Therefore, there is a possibility of cointegration in these variables. Then cointegration tests are conducted to examine whether there is a stable long-run equilibrium relationship between economic growth and embodied carbon in interregional trade. Popular cointegration tests for panel data include the Pedroni test, the Kao test, and the Fisher cointegration test, hence, in this research, the panel cointegration test is done using this three cointegration tests to examine the cointegration connection between variables, and the test results are presented in Table 3.

**Table 3:** Panel cointegration test results.

**Cointegration test**	**Statistic**	**Statistical value**	***P*-value**
Pedroni	Panel v	2.6427*	0.0596
	Panel rho	−1.9845	0.1724
	Panel ADF	−4.8963***	0.0011
	Panel PP	−3.1481**	0.0192
	Group rho	2.3599	0.7365
	Group ADF	−3.7216***	0.0048
	Group PP	−2.8952**	0.0359
Johansen Fisher	None	546.2***	0.0000
	At most 1	173.5***	0.0000
	At most 2	109.8**	0.0126
Kao	ADF	−5.6748***	0.0005

^*^, ^**^ and ^***^ represent passing the 10, 5, and 1% significance tests, respectively, allowing the original hypothesis to be rejected. The cointegration test equation includes an intercept term. The maximum lag order determined by the AIC criterion is 3.

The findings of the cointegration test suggest that in the long run, LnEC, LnGDP, and (LnGDP)^2^ converge, and there is a cointegration relationship between the non-stationary time series. Its equation regression residuals are smooth. On this basis, regression may thus be done.

### Regression results analysis

This article employs three estimating strategies based on data from 30 provinces from 2001 to 2019: mixed OLS, random-effects models, and fixed-effects models. The F-test rejects the original hypothesis since the F statistic corresponds to a *P* < 0.05. In addition, the Hausman statistic equates to a *P*-value of 0.0196 < 0.05. Excluding unobservable effects that do not change over time makes the selection of fixed effects more appropriate for model estimation. In this paper, the findings of the fixed-effects model estimation serve as the primary demonstration, while the random-effects models estimation and mixed OLS estimation serve as references. Table 4 displays the comprehensive estimation and test results.

**Table 4:** Model estimation results.

**Variable**	**OLS**	**FE**	**RE**
Constant	−18.3629**	−11.9814***	−12.5726***
LnPGDP	4.5769**	3.4805***	3.2688***
(LnPGDP)^2^	−0.1901**	−0.1718***	−0.1542**
R^2^	0.7588	0.9593	0.6041
F-statistic	406.5971	149.1692	49.5369
Turning point	e^12.04^ = 169,075.39	e^10.13^ = 25,084.36	e^10.59^ = 40,102.74
Curve shape	Inverted U	Inverted U	Inverted U
F test	135.6782 (Prob. = 0.0000)		
Hausman test	8.9903 (Prob. = 0.0196)		

*, ** and *** represent passing the 10%, 5%, and 1% significance tests, respectively, allowing the original hypothesis to be rejected. Real GDP per capita is obtained in constant 1978 prices in China.

As shown by the regression findings, all three-panel models pass a significance test with at least a 5% level of confidence and have a negative quadratic term and a positive linear item for economic growth. As a result, there is an inflection point for embodied carbon emissions in inter-regional trade, and the link between economic growth and embodied carbon emissions in inter-regional trade has an inverted U shape. The inflection point of the Kuznets curve could be computed using the formula for the vertex of a parabolic function. The inflection point of the embodied carbon emissions in inter-regional trade occurs at a GDP per capita of ¥25,084.36 in the fixed effects model in constant 1978 prices.

The economic significance of this estimation result is that embodied carbon emissions have a positive relationship with GDP per capita when GDP per capita is below ¥25,084.36. This is due to the fact that as the economy expands, the demand for fossil energy in industrial development, especially in the secondary sector, increases, making the implied carbon emissions in the energy-consumption-based industrial sector continue to increase. When the economic growth reaches the inflection point of ¥25,084.36, the environmental pollution caused by the embodied carbon emissions can be alleviated, so people gradually start to pay attention to energy saving and emission reduction. The effectiveness of emission reduction improvement can bring people the same economic benefits. When the GDP per capita exceeds ¥25,084.36, the embodied carbon emissions will gradually decrease as the economy grows. At this stage, people realize that although the high-energy consumption-based development model can achieve rapid economic growth, its damage to the ecological environment, especially climate change, cannot be ignored. In order to reverse this unfavorable situation, green energy and green technologies will be widely used. These energy and technological advances allow the economy to maintain a continuous growth trend while the embodied carbon emissions are gradually reduced.

### Peak prediction

In this paper, the GDP per capita turning point is computed using the inflection point indicated by the fixed-effects model in the regression findings. In the meantime, based on the average annual growth rate of real GDP per capita at constant prices in China in 1978, the growth rate of GDP per capita is divided into three scenarios: higher (7.85%), average (6.54%), and lower (5.39%), in order to estimate the time required for embodied carbon emissions in inter-regional trade to reach the peak and the year of appearance. The results of the calculations are reported in Table 5.

**Table 5:** Theoretical time for embodied carbon emissions in inter-regional trade to reach the peak.

	**PGDP growth**	**Year of peak**	**Required time**
	**rate (%)**		**(years)**
China	7.85	2030	7.56
	6.54	2034	11.83
	5.39	2037	14.75

Calculate the time needed to reach the peak using 2022 as the starting point.

The inflection points for the arrival of embodied carbon emissions in inter-regional trade calculated from three per capita GDP growth rates of high (7.85 percent), average (6.54 percent), and low (5.39 percent) occur in 2030, 2034, and 2037, respectively, according to Table 5. Under different GDP per capita growth rate assumptions, the peak paths of embodied carbon emissions in inter-regional trade vary significantly. Evidently, if the current rate of economic development and carbon emission reduction policies continue, meeting the target of reaching the peak of embodied carbon emissions in inter-regional trade by 2030 would be extremely challenging. Consequently, the government should implement suitable fiscal and tax policies, as well as commensurate incentives and regulatory measures. Such as providing tax incentives to companies with cleaner output and offering green low-interest loans to encourage companies to prioritize the research and implementation of energy-saving and emission-reduction technology. Regions with high carbon emissions should aggressively pursue industrial transformation and develop a green economy simultaneously.

The GDP growth rate per capita in each province is computed based on the average annual growth rate of real GDP per capita at constant prices in 1978 in each province. On this basis, the time necessary for embodied carbon emissions in inter-regional trade to reach the peak and the year when the peak is anticipated to occur are computed for each province. The results of the calculations are reported in Table 6.

**Table 6:** Theoretical time for embodied carbon emissions in inter-regional trade to reach the peak in each province of China.

**Province**	**PGDP growth rate (%)**	**Year of peak**	**Required time (years)**	**Province**	**PGDP growth rate (%)**	**Year of peak**	**Required time (years)**
Beijing	8.16	2019	-	Henan	8.24	2031	8.53
Tianjin	8.11	2022	-	Hubei	8.87	2028	5.46
Hebei	6.95	2034	11.45	Hunan	9.32	2030	7.07
Shanxi	7.92	2033	10.36	Guangdong	6.85	2026	3.39
Inner Mongolia	12.68	2024	1.73	Guangxi	8.71	2032	9.68
Liaoning	7.54	2027	4.89	Hainan	9.04	2033	10.63
Jilin	8.37	2028	5.61	Chongqing	8.37	2029	6.19
Heilongjiang	6.03	2036	13.99	Sichuan	9.53	2030	7.82
Shanghai	5.19	2020	-	Guizhou	10.68	2031	8.05
Jiangsu	8.68	2021	-	Yunnan	7.98	2036	13.97
Zhejiang	7.31	2024	1.58	Shaanxi	11.61	2026	3.25
Anhui	8.17	2032	9.49	Gansu	7.72	2037	14.48
Fujian	7.42	2026	3.65	Qinghai	9.88	2030	7.39
Jiangxi	8.55	2032	9.81	Ningxia	10.54	2027	4.85
Shandong	8.79	2026	3.74	Xinjiang	7.46	2035	12.21

Calculate the time needed to reach the peak using 2022 as the starting point.

As indicated in Table 6, due to disparities in economic condition, natural resource endowment, and energy consumption of high-carbon industries in each province, the time required to meet the carbon peak objective varies considerably. Based on the per capita GDP of each region, Beijing, Tianjin, Shanghai, and Jiangsu have already achieved their peak, thus the embodied carbon emissions would exhibit a falling trend as per capita GDP increases. These four regions' economic growth has been post-industrial, indicating that their industrial structure is more rational and that their energy intensity and energy structure offer advantages that are difficult to compare with those of other regions. Gansu reaches its inflection point in 2037, the latest anticipated among Chinese provinces. As a result of Gansu Province's industrial structure, which is dominated by heavy chemical industries, as well as its high energy consumption, high emissions, and resource-intensive economic growth model, resource and environmental disputes have become increasingly prominent. Combined with the low vegetation cover and weak carbon sink capacity of the ecosystem, the economic development of Gansu Province faces greater pressures of energy structure adjustment and industrial structure transformation than other regions, as well as a steeper path of energy conservation and emission reduction, making the task of achieving the carbon peak target challenging.

As can be observed, provinces in the eastern area achieve the peak first, with the majority appearing about 2025; followed by provinces in the central region, with the inflection point appearing around 2030; and finally, provinces in the western region reach the peak last. The eastern region, which has the highest level of economic development, relatively mature manufacturing and service industries, and relatively stringent environmental regulations, was the first to reach the embodied carbon emissions peak by shifting the burden of carbon emission reduction to other regions through the importation of primary products. As the ancient industrial bases in the northeast deteriorate, more energy businesses and industrial sectors are relocating to the center area. In addition, as a significant national supplier of energy, particularly electricity, the central region has accepted the embodied carbon emissions transfer from other regions and shifted some high energy-consuming and low value-added industries to the periphery in order to develop high-tech industries and service industries, thereby reducing the embodied carbon emissions. Hence, the peak time resides between the east and the west. The majority of western provinces are economically undeveloped, have an imperfect industrial basis. They are rich in natural resources and minerals, and are energy-intensive consumers. By manufacturing crude and energy-intensive primary goods to supply services for the upstream of the value chain, the industrial chain is relegated to the middle and bottom of the value chain, using more energy for less added value.

## Discussion of the Kuznets curve based on embodied carbon

### Stage division

**Figure 2:**
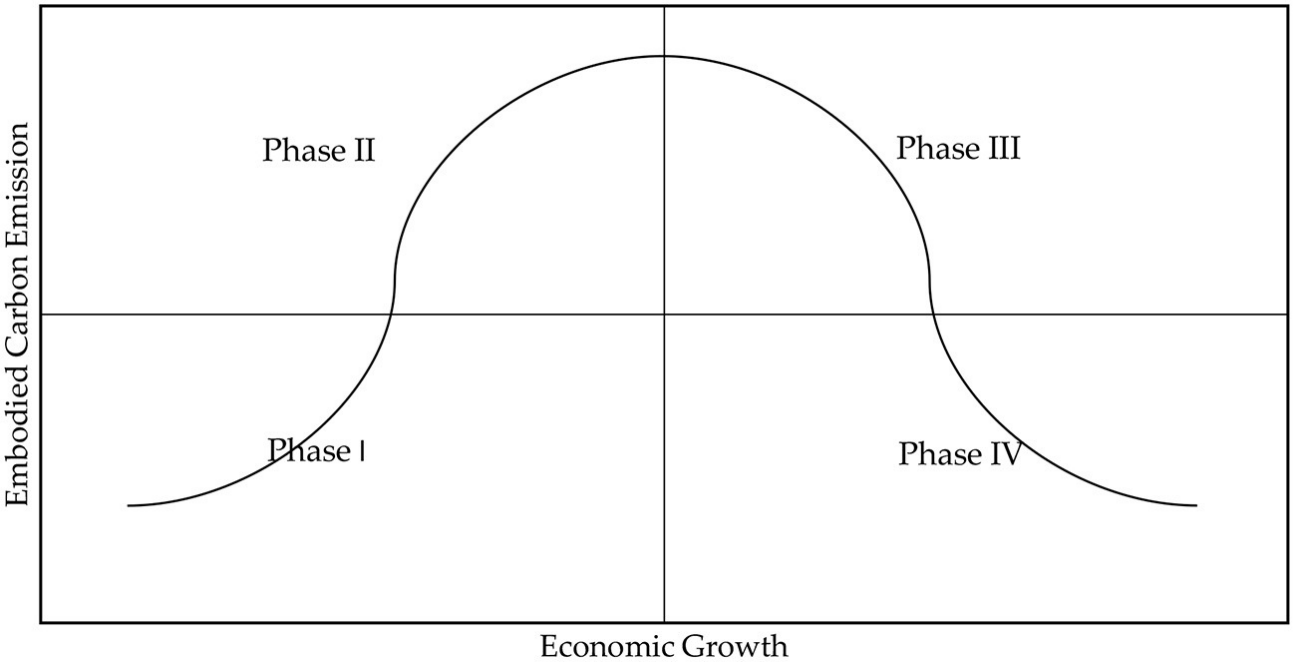
Economic growth and embodied carbon emission quadrant chart.

Not only does the environmental Kuznets curve indicate the actual link between economic growth and environmental quality, but it also discloses the key laws behind the empirical relationship between economic growth and environmental quality. Embodied carbon emissions in China's inter-regional trade also conform to the environmental Kuznets curve, and the conclusions obtained provide a theoretical foundation for the Chinese government to formulate emission reduction policies and fight the battle of energy conservation, emission reduction, and environmental management. In order to promote the development of the environmental Kuznets curve theory, this paper attempts to divide the environmental Kuznets curve with embodied carbon emissions in inter-regional trade as the independent variable into stages and investigate the varying performance of embodied carbon emissions in inter-regional trade at each stage of economic development, as depicted in Figure 2.

Each stage's features are mentioned below.

Stage I, with a low economy and low embodied carbon emission. During this stage, there is a high concentration of labor in the agricultural sector, but agricultural productivity is confined by land, and additional agricultural inputs are likely to be met with falling marginal returns, rendering agricultural income growth unsustainable. To increase revenue, employees will be encouraged to leave the agricultural sector and enter the non-agricultural sector. The non-agricultural sector, particularly the industrial sector, expands moderately but on a lower scale.

Stage II is characterized by a low economy and high embodied carbon emission. This stage strengthens the economy at the expense of the environment. For a longer length of time, resource-intensive and labor-intensive industries with low-value-added and high-carbon emission will dominate, and they will also absorb a portion of the transfer of high-polluting industries from other regions. At present time, energy consumption is still dominated by fossil energy, which has lower extraction and usage costs, while the use of clean energy is restricted by its technological level and has a negligible influence. It is burdened by increased embodied carbon emissions, a more rigid industrial structure, and an energy consumption pattern.

Stag III, high economy and high embodied carbon emission. The rapid economic growth and change from a secondary to a tertiary industry are characteristics of the development model of this stage. Additionally, due to the cyclical nature and lag in green technology development, it is unable to keep up with the rate of structural adjustment. As a result, its energy use efficiency level and low-carbon technology are unable to meet the requirements of the new development model and have a corresponding impact on embodied carbon emissions. This is reflected in the fact that the total embodied carbon emissions are still high overall but are trending downward.

Stage IV has a prosperous economy and minimal embodied carbon emission. At this stage, structural adjustment and technical progress are at a high level, which means that the rate of industrialization and embodied carbon emissions are continuously declining while the share of renewable energy is rising. In essence, a decoupling between economic development and embodied carbon emissions has been achieved, leading to the leadership of capital-intensive and technology-intensive industries with high added value but low carbon emissions and the formation of a more comprehensive low-carbon industrial chain. Additionally, the creation and use of clean energy have been improved along with industrial upgrading and technique improvement. However, this stage of development often has more technologically sophisticated regions, and the employment of low-carbon technology directly contributes to an increase in energy usage efficiency. Energy efficiency is directly improved by the deployment of low-carbon technology. These factors working together have caused embodied carbon emissions to decline to low levels.

### Regional comparison

The development of the environmental Kuznets curve of the embodied carbon emissions in inter-regional trade for China as a whole is examined. Based on the association between GDP per capita and embodied carbon emissions in inter-regional trade, the 1950's and 1980's represent the beginning of the first stage. China's industrial basis was poor, mostly focused on agricultural and by-product processing and extractive sectors, labor-intensive, and its total industrial level was extremely low. Both the total economic volume and the per capita level were incredibly low, and the economy as a whole was quite weak. In 1952, China's overall GDP represented a negligible part of the global economy, and in 1978 it had only reached 1.8%. Because industry is small and trade is underdeveloped, embodied carbon emissions in inter-regional trade are low.

The period between the reform and openness and the beginning of the 21st century represents the midpoint of the first stage. China's economy grew swiftly. A comprehensive and well-defined industrial structure was formed over time, and the country's ability to provide products and services increased significantly. However, technical research and development capacities were inadequate at the time, and a rudimentary development model was employed to boost economic strength mostly at the expense of the natural environment. During a period of tremendous economic expansion, embodied carbon emissions in China's inter-regional trade rose considerably.

The years 2000 to 2010 represent the last part of the first stage. China's economic structure witnessed a massive transformation from imbalanced to reasonably balanced, and economic development coordination was greatly improved. The industrial structure has shifted from simple to comprehensive categories, from predominantly light industry to the combined growth of light and heavy industry, and from a labor-intensive industry to a labor, capital and technology-intensive industry. Therefore, the overall embodied carbon emissions in inter-regional trade continue to rise, but the marginal effect of embodied carbon emissions in inter-regional trade is diminishing as industrial technology advances and pollution control mechanisms are strengthened.

The 2010's through the present constitute the second stage. The embodied carbon emissions in inter-regional trade in China will continue to rise as the economy develops, resulting in some environmental strain. However, China is also implementing effective control mechanisms to lessen the strain on embodied carbon emissions in inter-regional trade, and the quality of the local environment has improved.

Based on the strategic objective of peak carbon by 2030 and neutrality carbon by 2060, the Chinese government is continually boosting the level of low carbon technology and energy efficiency by investing more in research and talent development. Simultaneously, low-carbon technologies are employed to advance the development of a low-carbon economy in order to achieve the reciprocal advantages of a low-carbon economy and low-carbon technologies. China is anticipated to attain peak embodied carbon emissions in 2030, approaching the third stage of the Kuznets curve. By 2060, it enters the fourth stage, followed by a shift to a route with diminishing embodied carbon emissions.

By initiating the industrialization process first, Beijing, Tianjin, Shanghai, and Jiangsu have completed stages I and II of the environmental Kuznets curve of the embodied carbon emissions in inter-regional trade and are either in the transition phase between stages II and III or have already entered stage III. Beijing and Shanghai's embodied carbon emissions have exhibited a progressive decline, indicating they are entering stage III. Tianjin and Jiangsu's embodied carbon emissions slowly increase or remain stable, indicating that they are set to reach Stage III. Other emerging provinces are primarily at stage II of the environmental Kuznets curve of the embodied carbon emissions in inter-regional trade, while the least developed regions, including Heilongjiang, Gansu, and Xinjiang, are at stage I.

## Conclusions and policy recommendations

### Conclusions

This paper seeks to examine the relationship between environmental pollution and economic growth from the perspective of embodied carbon emissions in inter-regional trade, beginning with an accurate measurement of embodied carbon emissions in inter-regional trade, examining the dynamic evolution and spatial and temporal patterns of embodied carbon emissions in inter-regional trade, and concluding with an analysis of embodied carbon emissions in inter-regional trade and economic growth. Based on the environmental Kuznets curve theory, the link between economic growth and embodied carbon emission is then analyzed by subdividing the Kuznets curve into stages and concluding with a regional comparison of stages. The four findings that follow are drawn.

As interprovincial commerce develops, embodied carbon emissions in inter-regional trade transfer resulting from intra-provincial value-added transfer exhibit an upward trend from an aggregate standpoint. From the perspective of each region, the trade embodied carbon emissions in the eastern coastal region and the northwest region show an increasing trend, especially in the northwest. In contrast, the trade embodied carbon in the southern coastal region, Beijing-Tianjin region, and northeastern region exhibits a gradual increase or decrease in concentration.

There is a significant inverted U-shaped association in China between embodied carbon emissions in inter-regional trade and economic growth. According to three possible settings of the average annual growth rate of GDP per capita, the inflection point is estimated to occur in 2030, 2034, and 2037. These settings are higher, average, and lower. If the influence of government measures on carbon intensity reduction is taken into account, the inflection point can be achieved sooner.

Due to differences in per capita GDP growth rates, the time necessary to attain embodied carbon emissions in inter-regional trade and the anticipated year varies greatly by area. In the eastern area, the environmental Kuznets curve reaches its inflection point early, with the majority of provinces emerging about 2025; in the central region, the inflection point occurs around 2030; and in the western region, it emerges later.

Embodied carbon emissions environmental Kuznets curve can be divided into four stages, namely low economy and low embodied carbon emission → low economy and high embodied carbon emission → high economy and high embodied carbon emission → high economy and low embodied carbon emission. There is no necessary positive relationship between economic development and embodied carbon emission in interregional trade. Embodied carbon emission in interregional trade has its characteristics at different economic levels. Economic development cannot be developed across stages. However, the positive factors of reducing embodied carbon emission in interregional trade in economic development can be emphasized to shorten the time of stages II and III, and enter stage IV as soon as possible to achieve the ideal state of high economic level and low embodied carbon emission level.

### Policy recommendations

Based on the initial findings, the following suggestions are made:

Distinguished emission reduction policies by region. To reduce carbon emissions, for instance, the Beijing-Tianjin region and the eastern coastal region should develop high value-added, low energy consumption, and low emission industries, such as high precision industries and service industries; The central and western regions should develop forward-looking industries to gradually reduce carbon emissions while maximizing their energy advantages and ensuring national energy security. In the eastern area, economic revival is prioritized while carbon emission reduction and environmental preservation are also considered.

The disparities in the temporal convergence of the environmental Kuznets curve for embodied carbon emissions among China's provinces indicate they are disparate economic and technological growth. Therefore, when introducing various energy-saving and emission-reducing technologies and developing profitable industries, each province should be allowed to choose its priorities and determine its low-carbon development and export trade policies according to its level of economic and technological development.

## Data availability statement

The raw data supporting the conclusions of this article will be made available by the authors, without undue reservation.

## Author contributions

SM and TW: conceptualization, methodology, validation, formal analysis, investigation, and project administration. SM: software, resources, data curation, writing—original draft preparation, writing—review and editing, and visualization. TW: supervision and funding acquisition. All authors have read and agreed to the published version of the manuscript.

## Funding

This research was funded by the Guizhou Province Science and Technology Fund Project, grant number ZK[2021] 275, the Guizhou Province Philosophy and Social Science Planning Joint Fund Project, grant number 18GZLH03, the Major Scientific Research Projects of Key Liberal Arts Disciplines and Characteristic Disciplines in Guizhou University, grant number GDZT201702, and the Guizhou Province Postgraduate Research Fund, grant number YJSKYJJ[2021] 017.

## Conflict of interest

The authors declare that the research was conducted in the absence of any commercial or financial relationships that could be construed as a potential conflict of interest.

## Publisher's note

All claims expressed in this article are solely those of the authors and do not necessarily represent those of their affiliated organizations, or those of the publisher, the editors and the reviewers. Any product that may be evaluated in this article, or claim that may be made by its manufacturer, is not guaranteed or endorsed by the publisher.
